# NSAIDs, analgesics, antiplatelet drugs, and decline in renal function: a retrospective case-control study with SIDIAP database

**DOI:** 10.1186/s40360-024-00771-5

**Published:** 2024-08-28

**Authors:** Sara Bonet-Monné, Cristina Vedia Urgell, M. José Pérez Sáez, Oriol Cunillera Puertolás, José Miguel Baena-Díez, Julio Pascual, Cristina Orive Lago, Jordi Rodriguez Ruiz, Betlem Salvador Gonzalez, Rosa Morros Pedrós

**Affiliations:** 1 Servei d’Atenció Primària Baix Llobregat Centre, Institut Català de la Salut, Cornellà de Llobregat, Spain; 2 Fundació Institut Universitari per a la Recerca a l’Atenció Primària de Salut Jordi Gol i Gurina (IDIAPJGol), Barcelona, Spain; 3 Servei d’Atenció Primària Barcelonès Nord i Maresme, Institut Català de la Salut, Badalona, Spain; 4 Servei de Nefrologia, Hospital del Mar, Barcelona, Spain; 5 Unitat de Suport a la Recerca (USR), Atenció Primària Metropolitana Sud, Institut Català de la Salut – IDIAPJGol, L’Hospitalet del Llobregat, Barcelona, Spain; 6 Servei Atenció Primària Esquerra, CAP La Marina, Institut Català de la Salut, Barcelona, Spain; 7 Servicio de Nefrologia y del programa de Trasplante Renal, Hospital 12 de Octubre, Madrid, Spain; 8 Atenció Primària Metropolitana Sud, CAP El Castell, Institut Català de la Salut, Castelldefels, Spain; 9 Unitat d’estudi del Medicament, Fundació Institut Universitari per a la Recerca a l’Atenció Primària de Salut Jordi Gol i Gurina (IDIAPJGol), Barcelona, Spain

**Keywords:** Case control study, Anti-inflammatory agents, Analgesics, Platelet aggregation inhibitors, Glomerular filtration rate

## Abstract

**Introduction:**

We aim to explore the association between NSAIDs consumption, Symptomatic Slow Action Drugs for Osteoarthritis (SYSADOA), analgesics, and antiplatelet drugs, and decline in renal function by estimated Glomerular Filtration Rate (eGFR).

**Methods:**

We performed a case-control study using the SIDIAP database in Catalonia. We considered defined cases, patients with an eGFR value ≤ 45 ml/min/1.73 m^2^ in the period 2010–2015 with a previous eGFR value ≥ 60, and no eGFR ≥ 60 after this period. Controls had an eGFR ≥ 60 with no previous eGFR < 60. Five controls were selected for each case, matched by sex, age, index date, Diabetes Mellitus and Hypertension. We estimated Odds Ratios (OR, 95% Confidence Intervals) of decline in renal function for drugs group adjusting with logistic regression models, by consumption measured in DDD. There were *n* = 18,905 cases and *n* = 94,456 controls. The mean age was 77 years, 59% were women. The multivariate adjusted model showed a low risk for eGFR decline for NSAIDs (0.92;0.88–0.97), SYSADOA (0.87;0.83–0.91) and acetaminophen (0.84;0.79–0.89), and an high risk for metamizole (1.07;1.03–1.12), and antiplatelet drugs (1.07;1.03–1.11). The low risk in NSAIDs was limited to propionic acid derivatives (0.92;0.88–0.96), whereas an high risk was observed for high doses in both acetic acid derivatives (1.09;1.03–1.15) and Coxibs (1.19;1.08–1.30). Medium and high use of major opioids shows a high risk (1.15;1.03–1.29). Triflusal showed high risk at medium (1.23;1.02–1.48) and high use (1.68;1.40–2.01).

**Conclusion:**

We observed a decline in renal function associated with metamizole and antiplatelet agent, especially triflusal, and with high use of acetic acid derivates, Coxibs, and major opioids. Further studies are necessary to confirm these results.

**Supplementary Information:**

The online version contains supplementary material available at 10.1186/s40360-024-00771-5.

## Introduction

Chronic kidney disease (CKD), characterized by chronic kidney damage resulting in reduced blood filtration, is a relatively frequent pathology growing worldwide due to increased life expectancy and the increase in risk factors associated with CKD, such as hypertension (HT) and type 2 diabetes mellitus (DM) [1].

The prevalence of CKD in Spain, defined by a reduced eGFR < 60 ml/min/1.73 m^2^ or an increased and persistent albumin/creatinine ratio ≥ 30 mg/g), is 15.1% higher in men than in women (23.1% vs. 7.3%), and increases with age to 37.3% in subjects ≥ 65 years [2]. Approximately 6.8% of the population has an eGFR of less than 45 ml/min, and 0.3% of the population has an eGFR of less than 30 ml/min.30 ml/min [3].

In Catalonia, in the period from 2012 to 2018, there has been an increase in both the prevalence and incidence of kidney replacement therapies, the number of transplants performed, and mortality associated with end-stage kidney disease [4].

The relationship between analgesic consumption and the appearance of CKD—known as analgesic nephropathy—is well established, although there is discordant data regarding the effect and magnitude of the different analgesics studied [5–14].

The possible effect of non-steroidal anti-inflammatory drugs (NSAIDs) on initial development or progression of CKD has been less studied. In this regard, a meta-analysis published in 2013 showed no relationship between the use of NSAIDs and CKD, except in the case of taking high doses of NSAIDs that decline in renal function [15]. For selective cyclooxygenase-2 inhibitors (Coxibs) there is still less information from medium and long-term studies on their possible nephrotoxicity. An association has been identified between both nonselective NSAIDs and Coxibs use and an increase in blood pressure, especially in patients with a history of HT. This effect could be a contributing factor in the long-term development of CKD [16–19].

There are no data for other analgesics such as metamizole or Symptomatic Slow Action Drugs for Osteoarthritis (SYSADOA) and development of CKD; however, due to the mechanism of action, SYSADOA could not have renal effect. Similarly, there is very limited data for opioids. In relation to antiaggregants the data are limited [20–26], a clinical trial with acetyl salicylic acid (ASA) in patients with chronic kidney disease and DM [23] did not show any effect on the decline in renal function, but a meta-analysis pointed to a possible protective effect [22].

Several factors hinder understanding of analgesic or NSAID nephropathy. Most studies based CKD status on serum creatinine, not eGFR, and they are primarily focused on advanced CKD or on renal substitute treatment, excluding the frequent mild- moderate phases usually attended in primary care. In these early stages, restrictions on the prescription of NSAIDs and analgesics are less frequent.

Given this lack of knowledge, the objective of this study is to explore the associations between decline in renal function and the use of NSAIDs (non-selective or Coxibs), SYSADOA, analgesics (acetaminophen, metamizole and opioids), and/or antiplatelet drugs. It also aims to establish whether there is a dose-effect relationship in terms of cumulative dose or time of use.

## Materials and methods

We performed a case-control study using the Information System for the Development of Research in Primary Care (SIDIAP), which incorporates the electronic health records from primary care (ECAP) at the “Institut Català de la Salut” (ICS), the main health provider in Catalonia and other data sources such as billing in the pharmacy offices of the Catalan Health Service (CATSALUT) [27, 28]. The ICS database contains longitudinal information from 2006 onwards from the ECAP clinical station of 274 primary care teams in Catalonia, with an assigned population of 5,835,000 patients (80% of the Catalan population). Information comprises patients’ demographic data (sex, age), health problems coded according to the “International Statistical Classification of Diseases and Related Health Problems, 10th Revision (ICD-10) [29], clinical visits to primary care centers, results of analytical and other complementary tests, and records of all medication prescribed and dispensed by the Catalan national health system.

### Participants

The target population consisted of individuals aged over 17 who were registered in SIDIAP, had attended a public primary care center, and who had a CKD-EPI or creatinine registry during the period from 2010 to 2015. Attendance was defined as a minimum of 2 visits to a primary care center (to be seen by a family physician and/or a nurse) in the 3 years prior to the index date selection of both cases and controls.

Patients with fewer than two visits in the last three years before the index data during the study period were excluded, in addition to those with previous primary renal diseases or other diseases which high risk of CKD, such as renal conditions, rheumatological diseases, oncological pathology (both due to the possible risk of the oncological process and the possibility of having received treatment with nephrotoxic drugs) and patients with organ transplants (Supplementary Table 1).

### Case/control definition

A patient was considered a case, if he or she had an eGFR value (estimated by the CKD-EPI formula) ≤ 45 ml/min/1.73 m^2^ [30] (index date) in the period between 2010 and 2015 (with this value date considered as the Index Date) with a previous eGFR value ≥ 60, and no eGFR ≥ 60 afterwards.

A patient was considered a control if he or she had an eGFR ≥ 60 (index date) in the period between 2010 and 2015 with no previous eGFR < 60. Moreover, no variation in eGFR values > 20% between 2005 and 2010 was required.

Five controls were randomly selected for each case through a density-based sampling method matched by sex, age (± 2 years), Index Date (± 1 year), diagnosis of DM and HT in the study period, and time of two laboratory tests (0–1 year, 1–2 years, or 2 or more years).

### Variables

We collected data on age, sex, smoking status (nonsmoker, former smoker, and current smoker), Charlson comorbidity index, HT, DM, heart failure, arteriosclerotic disease, atrial fibrillation, hypercholesterolemia, anemia, hyperuricemia (Supplementary Table 1) and concomitant drug use related to renal involvement (Supplementary Table 2).

We considered the use and pattern of use of the study drugs: NSAIDs, SYSADOA, analgesics (acetaminophen, metamizole and opioids), and/or antiplatelet drugs. We defined daily doses (DDDs) [31] billed until the index date per patient for each study group and drug, according to the codes of the “Anatomical Therapeutic Chemical Classification System” (Supplementary Table 2).

Three exposure variables were generated for drug use. Firstly, patients were classified as drug users if there was a minimum withdrawal of three packages of the same active substance from the pharmacy in the period studied. We selected 3 or more prescriptions withdrawn from pharmacies to ensure adherence to treatment by using dispensing data and not having actual consumption data. Secondly, cumulative DDD was calculated for each patient, classifying patients into “No use” or, according to tertiles of cumulative DDD, “Low Use”, “Medium use” or “High use” into a dosage variable. Lastly, the pattern of use was defined as “No use”, “Current or recent use” (having received at least one prescription in the 12 months prior to the index date) or “Remote use” (drug received earlier than 12 months before the index date).

### Statistical methods

Baseline characteristics between cases and controls were compared. Quantitative variables were described using mean and standard deviation, and median and interquartile range. Qualitative variables were described using absolute and relative frequencies.

Chi-square tests were used to compare qualitative variables between cases and controls, while Mann-Whitney U tests were used for quantitative variables. Odds Ratios (ORs), with 95% Confidence Intervals (CIs) for decline in renal function (indicating the case group) were estimated by adjusting logistic regression models.

ORs were estimated both unadjusted (from a logistic regression model using the drug) and adjusted from a multivariate logistic regression model (adjusting for the index data year, comorbidities [Supplementary Table 1] and concomitant drugs [Table 1]).

**Table 1:** Baseline characteristics of the case-control population

		Case group (*n* = 18905)	Control group (*n* = 94456)	Odds ratio
Index date year				
	2010	2242 (11.9%)	10,186 (10.8%)	Ref
	2011	2289 (12.1%)	12,185 (12.9%)	0.85 (0.80, 0.91)
	2012	2872 (15.2%)	13,754 (14.6%)	0.95 (0.89, 1.01)
	2013	2980 (15.8%)	15,325 (16.2%)	0.88 (0.83, 0.94)
	2014	3731 (19.7%)	19,646 (20.8%)	0.86 (0.82, 0.91)
	2015	4791 (25.3%)	23,360 (24.7%)	0.93 (0.88, 0.99)
Age	Median [IQR]	80.00 [72.00, 85.00]	79.00 [72.00, 84.00]	1.01 (1.00, 1.01)
	Mean (SD)	77.76 (10.90)	77.21 (10.57)	1.01 (1.00, 1.01)
Sex	Women	11,292 (59.7%)	56,412 (59.7%)	Ref
	Men	7613 (40.3%)	38,044 (40.3%)	1.00 (0.97, 1.03)
Smoking status				
	Non smoker	13,711 (72.5%)	70,936 (75.1%)	Ref
	Former smoker	3648 (19.3%)	16,821 (17.8%)	1.12 (1.08, 1.17)
	Current smoker	1546 (8.2%)	6699 (7.1%)	1.19 (1.13, 1.27)
Smoking	Former + Current	5194 (27.5%)	23,520 (24.9%)	1.14 (1.10, 1.18)
Charlson				
	No or low comorbidity (0 to 2 points)	14,272 (75.5%)	87,590 (92.7%)	Ref
	High comorbidity (3 or more points)	4633 (24.5%)	6866 (7.3%)	4.14 (3.97, 4.32)
Atherosclerotic Cardiovascular Disease		5936 (31.4%)	18,345 (19.4%)	1.90 (1.83, 1.97)
Heart Failure		3795 (20.1%)	5232 (5.5%)	4.28 (4.09, 4.48)
Atrial fibrillation		3752 (19.9%)	9007 (9.5%)	2.35 (2.25, 2.45)
Hypercholesterolemia		12,472 (66.0%)	66,459 (70.4%)	0.82 (0.79, 0.84)
Anemia		9987 (52.8%)	19,595 (20.8%)	4.28 (4.14, 4.42)
Hyperuricemia		11,082 (58.6%)	17,835 (18.9%)	6.09 (5.89, 6.29)
Diabetes		7458 (39.5%)	34,974 (37.0%)	1.11 (1.07, 1.14)
Hypertension Evolution		15,427 (81.6%)	73,805 (78.1%)	1.24 (1.19, 1.29)
**Concomitant drug use**				
Allopurinol		1638 (8.7%)	2947 (3.1%)	2.95 (2.77, 3.14)
Febuxostat		32 (0.2%)	15 (0.0%)	10.68 (5.88, 20.27)
Calcium channel antagonists		5173 (27.4%)	20,106 (21.3%)	1.39 (1.34, 1.44)
Angiotensin-converting-enzyme inhibitors		8762 (46.4%)	34,176 (36.2%)	1.52 (1.48, 1.57)
Angiotensin II receptor blocker		5011 (26.5%)	17,009 (18.0%)	1.64 (1.58, 1.70)
Loop diuretics		7056 (37.3%)	10,358 (11.0%)	4.84 (4.67, 5.01)
Thiazides		3427 (18.1%)	16,911 (17.9%)	1.02 (0.98, 1.06)
Beta blockers		5498 (29.1%)	15,106 (16.0%)	2.15 (2.08, 2.23)
Calcium		2140 (11.3%)	11,334 (12.0%)	0.94 (0.89, 0.98)
Statins		8116 (42.9%)	38,035 (40.3%)	1.12 (1.08, 1.15)
Proton-pump inhibitors		11,716 (62.0%)	44,861 (47.5%)	1.80 (1.75, 1.86)
Lithium		39 (0.2%)	80 (0.1%)	2.44 (1.65, 3.55)
Bisphosphonates		1115 (5.9%)	6068 (6.4%)	0.91 (0.85, 0.98)

The statistical analysis was performed with the R statistical package (https://cran.r-project.org/).

## Ethical issues

All procedures were in accordance with applicable regulation on clinical research and data protection, and with ethical standards of the 1964 Helsinki Declaration and its later amendments, or comparable ethical standards. The study protocol was approved by the Ethics Committee for Clinical Research of IDIAP Jordi Gol (reference no. P14/072). Exemption of informed consent was considered from the participating individuals and the study was based on pseudonymized data according on data protection law.

## Results

**Fig. 1:**
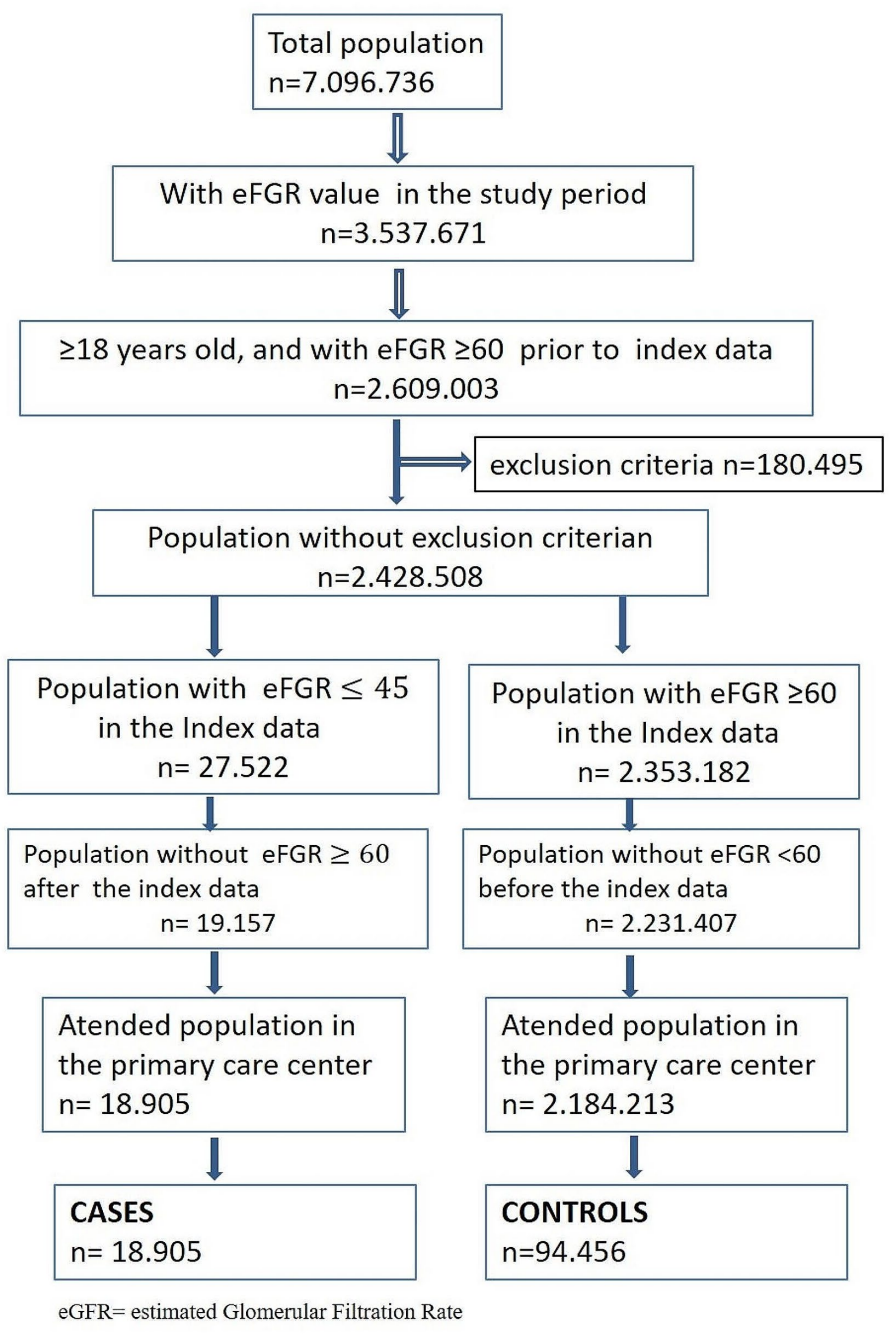
Study flow-chart

Within the study cohort, 18,905 cases were identified and matched with 94,456 controls (see flow-chart in Fig. 1).

The mean age of the sample was approximately 77 years, 60% were women, 79% had HT, and 37% had DM. The characteristics of the sample for cases and controls are described in Table 1.

We observed that the prevalence of other cardiovascular risk factors is higher in cases than in controls, except for hypercholesterolemia. Moreover, the use of concomitant medications is also higher in cases than in controls, except for calcium supplements and bisphosphonates, which are lower, and for diuretics, which do not indicate differences in levels.

The use of the different groups of study drugs and OR (crude and adjusted risks of decline in renal function) is shown in Table 2. The higAngiotensin-convertinghest use in both cases and controls can be seen for acetaminophen, followed by NSAIDs and opioids.

**Table 2:** Use to the different study groups of drugs by decline in renal function, and association of drug use and renal function impairment (results from unadjusted and adjusted logistic regression models)

	Case group (*n* = 18905)	Control group (*n* = 94456)	Unadjusted odds ratio	*p*-value	Adjusted odds ratio*	*p*-value
Nonsteroidal anti-inflammatory drugs	15,724 (83.2%)	79,015 (83.7%)	0.97 (0.93, 1.01)	0.105	0.92 (0.88, 0.97)	0.002
Slow action drugs for Osteoarthritis	3024 (16.0%)	17,497 (18.5%)	0.84 (0.80, 0.87)	< 0.001	0.87 (0.83, 0.91)	< 0.001
Opioids	10,607 (56.1%)	49,930 (52.9%)	1.14 (1.11, 1.18)	< 0.001	0.98 (0.95, 1.03)	0.445
Acetaminophen	16,902 (89.4%)	82,895 (87.8%)	1.18 (1.12, 1.24)	< 0.001	0.84 (0.79, 0.89)	< 0.001
Metamizole	8186 (43.3%)	35,828 (37.9%)	1.25 (1.21, 1.29)	< 0.001	1.07 (1.03, 1.12)	< 0.001
Antiplatelet drugs	9688 (51.3%)	37,273 (39.5%)	1.61 (1.56, 1.66)	< 0.001	1.07 (1.03, 1.11)	0.002

*Adjusted by; Index data year, Charlson index, Atherosclerotic Cardiovascular Disease, Heart Failure, Atrial fibrillation, Hypercholesterolemia, Anemia, Hyperuricemia, Diabetes Mellitus, Smoking habit and concomitant drugs (Allopurinol, Febuxostat, Calcium channel antagonists, Angiotensin-converting-enzyme inhibitors, Angiotensin II receptor blocker, Loop diuretics, Thiazides, Beta blockers, Calcium, Statins, Proton-pump inhibitors, Lithium, Bisphosphonates)

### Risk of decline in renal function depending on the analgesic family drug

When applying the multivariate adjusted model (Table 2), a low risk is described for NSAIDs (OR 0.92; 95%CI, 0.88–0.97), SYSADOA (OR 0.87; 95%CI,0.82–0.91) and acetaminophen (OR 0.84; 95%CI, 0.79–0.89), a high risk for metamizole (OR 1.07; 95%CI, 1.03–1.12) and antiplatelet drugs (OR 1.07; 95%CI, 1.03–1.11) and no effect for opioids (OR 0.98; 95%CI, 0.95–1.03).

When analyzing the subgroups, (Table 3 and Supplementary Fig. 1), within the NSAIDs the low risk was limited to propionic acid-derivatives (PAD) (OR 0.92; 95%CI, 0.88–0.96). For the opioids, a high risk of decline in renal function appears to be associated only with major opioids (OR 1.15; 95%CI, 1.08–1.23).

**Table 3:** Use to the different study sub groups of drugs by decline in renal function, and association of drug use and renal function impairment (results from adjusted logistic regression models)

	Case group (*n* = 18905)	Control group (*n* = 94456)	Adjusted *R* (95%CI)*	*p*-value
**NSAID**				
Acetic acid derivatives NSAIDs^a^	9683 (51.2%)	46,397 (49.1%)	1.01 (0.97, 1.05)	0.555
Enolic acid (oxicam) derivatives NSAIDs^b^	1941 (10.3%)	9638 (10.2%)	0.99 (0.93, 1.05)	0.626
Propionic acid derivatives NSAIDs^c^	14,006 (74.1%)	71,580 (75.8%)	0.92 (0.88, 0.96)	< 0.001
Coxibs NSAIDs^d^	2382 (12.6%)	11,881 (12.6%)	1.01 (0.95, 1.07)	0.868
Other NSAIDs^e^	372 (2.0%)	1794 (1.9%)	0.93 (0.82, 1.07)	0.314
**Opioids**				
Major opioids	1885 (10.0%)	5786 (6.1%)	1.15 (1.08, 1.23)	< 0.001
Minor opioids	10,337 (54.7%)	49,097 (52.0%)	0.97 (0.93, 1.01)	0.149
**Antiplatelet Drugs**				
ASA alone	9422 (49.8%)	36,235 (38.4%)	1.05 (1.01, 1.09)	0.026
Associated ASA	74 (0.4%)	122 (0.1%)	1.44 (1.00, 2.04)	0.046
Triflusal	590 (3.1%)	2052 (2.2%)	1.29 (1.16, 1.44)	< 0.001

^a^Acetic acid derivatives NSAIDs (AAD): indomethacin, sulindac, tolmetin, diclofenac, alclofenac, proglumethacin, ketorolac, aceclofenac

^b^Enolic acid (oxicam) derivatives NSAIDs: piroxicam, tenoxicam, lornoxicam, meloxicam

^c^Propionic acid derivatives NSAIDs (PAD): ibuprofen naproxen, ketoprofen, flurbiprofen, dexibuprofen, dexketoprofen

^d^Coxibs NSAIDs: celecoxib, etoricoxib

^e^Other NSAIDs: Isonixina, nabumetone, niflumic acid, morniflumato, oxaceprol, mefenamic acid, phenylbutazone, oxyphenbutazone

*Adjusted by; Index data year, Charlson index, Atherosclerotic Cardiovascular Disease, Heart Failure, Atrial fibrillation, Hypercholesterolemia, Anemia, Hyperuricemia, Diabetes Mellitus, Smoking habit and concomitant drugs (Allopurinol, Febuxostat, Calcium channel antagonists, Angiotensin-converting-enzyme inhibitors, Angiotensin II receptor blocker, Loop diuretics, Thiazides, Beta blockers, Calcium, Statins, Proton-pump inhibitors, Lithium, Bisphosphonates)

In relation to antiplatelet agents, all the subgroups present a high risk of decline in renal function: ASA alone (OR 1.05; CI95%, 1.01–1.09), ASA associations (OR 1.44; CI95%, 1.00- 2.04), and triflusal (OR 1.29; CI95%, 1.16–1.44).

### Risk of decline in renal function depending on previous consumption (DDD)

Data are also analyzed according to the degree of use by DDD tertiles (Table 4). The low risk of NSAIDs is limited to low (OR 0.92; 95%CI, 0.87–0.98) or medium use (OR 0.91; 95%CI, 0.85–0.96). Within the NSAID subgroup (Supplementary Table 3) the low risk is observed at any dose use of propionic acid derivatives, while an high risk is detected for high doses both in acetic acid derivatives (AAD) (OR 1.09; 95%CI, 1.03–1.15) and Coxibs (OR 1.19; 95%CI, 1.08–1.30).

**Table 4:** Multivariate regression model on adjusted decline in renal function by cumulative doses of drugs (categorized in DDD tertiles)

		Adjusted OR (95%CI)*	*p*-value
Nonsteroidal anti-inflammatory drugs	No use	(ref.)	
	Low use	0.92 (0.87, 0.98)	0.005
	Medium use	0.91 (0.85, 0.96)	0.002
	High use	1.02 (0.95, 1.09)	0.604
Slow Action Drugs for Osteoarthritis	No use	(ref.)	
	Low use	0.89 (0.82, 0.96)	0.003
	Medium use	0.83 (0.76, 0.90)	< 0.001
	High use	0.86 (0.79, 0.93)	< 0.001
Opioids	No use	(ref.)	
	Low use	0.94 (0.89, 0.99)	0.027
	Medium use	1.00 (0.95, 1.06)	0.973
	High use	1.06 (1.01, 1.13)	0.034
Acetaminophen	No use	(ref.)	
	Low use	0.91 (0.85, 0.97)	0.004
	Medium use	0.82 (0.76, 0.87)	< 0.001
	High use	0.70 (0.65, 0.76)	< 0.001
Metamizole	No use	(ref.)	
	Low use	1.05 (0.99, 1.11)	0.104
	Medium use	1.07 (1.01, 1.13)	0.022
	High use	1.14 (1.08, 1.21)	< 0.001
Antiplatelet Drugs	No use	(ref.)	
	Low use	1.17 (1.11, 1.23)	< 0.001
	Medium use	1.09 (1.03, 1.15)	0.004
	High use	0.94 (0.89, 1.00)	0.042

*Adjusted by; Index data year, Charlson index, Atherosclerotic Cardiovascular Disease, Heart Failure, Atrial fibrillation, Hypercholesterolemia, Anemia, Hyperuricemia, Diabetes Mellitus, Smoking habit and concomitant drugs (Allopurinol, Febuxostat, Calcium channel antagonists, Angiotensin-converting-enzyme inhibitors, Angiotensin II receptor blocker, Loop diuretics, Thiazides, Beta blockers, Calcium, Statins, Proton-pump inhibitors, Lithium, Bisphosphonates)

Within SYSADOA and acetaminophen, the low risk effect is maintained for all degrees of DDD use (Table 4, Supplementary Fig. 2).

For the opioids group, a high risk appears with high use (OR 1.06; 95%CI, 1.01–1.13) in the case of major opioids (Supplementary Table 3).

In addition, medium and high use of metamizole is associated with a high risk (OR 1.07; 95% CI 1.01–1.13; and OR 1.14; 95%CI 1.08–1.21, respectively) (Table 4).

For the antiplatelet drugs, high risk occurs at low (OR 1.17; 95%CI, 1.11–1.23) and medium use (OR 1.09; 95%CI, 1.03 − 1.15), with low risk at high use (OR 0.94; 95%CI 0.89–1.00). The same pattern was maintained for ASA alone (Supplementary Table 3). In contrast, triflusal shows a high risk at medium (OR 1.23; 95%CI, 1.02–1.48) and high use (OR 1.68 95%CI, 1.40–2.01).

### Risk of decline in renal function depending on the time of consumption

Supplementary Tables 4 and 5 show the analysis according to recent or remote use. In the NSAIDs group, acetic acid (OR 1.17; 95%CI, 1.11–1.30) and Coxibs (OR 1.14; 95%CI, 1.03–1.27) shows a high risk within recent use. This is also the case for metamizole (OR 1.22; 95%CI, 1.15–1.29). For the opioids group, the high risk occurs only for major opioids in both recent and remote use (OR 1.16; 95% CI, 1.06–1.27, and OR 1.11; 95%CI, 1.01–1.22 respectively).

For antiplatelet agents an increased risk is observed for both recent and remote use (OR 1.08; 95%CI, 1.03–1.13 and OR 1.06; 95%CI, 1.00- 1.13 respectively). AAS alone shows a high risk for both categories (OR 1.06; 95%CI, 1.012–1.11 and OR 1.06; 95%CI, 1.00- 1.13 respectively), while for associated AAS and triflusal the high risk is limited to recent use (OR 1.64; 95%CI, 1.07–2.50, and OR 1.89; 95%CI, 1.58–2.25 respectively).

## Discussion

In our study there was no decline in renal function related to NSAIDs, but it appeared in highest cumulative doses (DDD) of acetic acid derivatives NSAIDs and Coxibs. A high risk of decline in renal function was identified for metamizole, antiplatelet agents and high dose of major opioids.

Although our study does not show a high risk of decline in renal function with overall NSAID use, results that would be consistent with other studies that have not associated these drugs with the development of chronic kidney disease [13–15], there are more studies that show an unfavorable effect of NSAIDs on renal function [5, 6, 9–12]. Thus, NSAIDs have been associated with renal failure [6, 9, 11, 12], chronic kidney disease [5] or end-stage renal disease [10]. It should be noted that our study does show a risk of decline in renal function with high NSAID use, a result that would be consistent with the data from the meta-analysis by Nderitu et al. [15] which also found no overall relationship between NSAID use and chronic kidney disease, except in the case of high exposure to NSAIDs, where it would be associated with decline in renal function [15].

Our results would therefore support current recommendations to avoid these drugs in patients with chronic kidney disease. However, this “no risk” is limited to propionic acid-derivatives for all the different analyses, such as dose exposure or pattern of use, which is consistent with a better safety profile (digestive and cardiovascular security) [32–35] of this subgroup of NSAIDs.

Moreover, a high risk is observed for high exposure in both acetic acid derivatives and Coxibs. This aligns with studies that describe kidney damage [13, 15]. For the Coxibs, this could be slightly higher, perhaps due to the effect of increased blood pressure described principally for this group [19], and especially in response to etoricoxib [18].

In our study, acetaminophen has a low risk of decline in renal function. These results align with other studies in which there was no associated risk of renal impairment [6, 9, 10, 14]; however, they also diverge from other studies [5, 7, 8, 12].

In the present analysis, metamizole shows a consistent high risk of decline in renal function (globally, for medium and high doses and recent exposition). Currently this drug tends to be used as an alternative to NSAIDs in patients with chronic kidney disease or hypertension.

There is limited information about a possible risk of metamizole, as it is only used in a few countries. Metamizole pharmacological effects seem to be partly mediated by interference with prostaglandin synthesis via inhibition of cyclooxygenases, [36–37], and use of high doses of this drug may prolong vasopressor therapy and could be an independent risk factor for acute kidney failure in intensive care units [38].

Previous studies on the safety of SYSADOA have not shown cardiovascular or gastrointestinal adverse effects of NSAIDs. According to our results, there are no adverse renal effects with chronic use [39].

For the opioids group, we observed a high risk only for major opioids, which seems to be associated with high use, both recent and remote use. Although it has been widely described that opioids need to be readjusted if renal function is severely impaired, we have not found information about decline in renal function due to these drugs.

Although the drug itself could explain this, it could also be a consequence of a possible bias in case population, as major opioids can be used as an alternative to NSAIDs in patients with higher and more severe comorbidity, which is also associated with chronic kidney disease.

Regarding antiplatelet drugs, the results show a high risk of decreasing eGFR observed for all antiplatelet agents, both for recent and remote use.

Interestingly, there is no risk in the consumption of high cumulative doses of ASA, a treatment that tends to be higher in CKD patients, which may indicate that it is not the drug itself posing the risk, but rather a possible confounding factor. We are especially concerned about triflusal data, a drug with a high level of use in our country.

In contrast in a Cochrane systematic review [40], the user of antiplateled drugs did not shows a high risk of decline in renal function. More recently, a systematic review showed that the use of antiplatelet therapy in CKD patients did not slow the rate of eGFR decline (MD, 0.15 mL/1.73 m^2^/year; 95% CI, −0.89 to 1.20; I2 = 40.8%), and had no effect on kidney failure events (OR, 0.87; 95% CI, 0.32–1.55) [41]. There were no data comparing different antiplatelet agents. The results were similar in the clinical randomized controlled trial conducted by Sandory to evaluate low-dose aspirin (81 mg or 100 mg daily) or aspirin-free group, as primary treatment prevention of CVD in patients with type 2 diabetes. They found that long-term low-dose aspirin does not affect eGFR or positive urine dipstick albumin in patients with type 2 diabetes [23].

In Fored’s retrospective case-control study of the relationship between analgesic use and kidney function, there was a high risk related to AAS at analgesic doses (OR 2.5) but not at antiplatelet doses [8].

On the other hand, the progressive aging of the population in recent decades has been associated with an increase in osteoarticular pathologies, frequently accompanied by chronic consumption of anti-inflammatory drugs and analgesics [42]. This may have contributed to the high prevalence of CKD in older people. Although in Spain [43, 44] and Catalonia the consumption of NSAIDs has decreased in recent years, local data show a 45% increase in metamizole use from 2014 to 2020 (2.0 DHD and 2.9 DHD, respectively).

Considering the arguments provided above, we consider that further studies would be necessary to confirm this decline in renal function, especially in relation to metamizole, major opioids and triflusal. Although the risk described in our study related to metamizole could be marginal, as it is one of the drugs frequently used as an alternative to NSAIDs in our country, this risk could be affecting many patients. With regard to triflusal, we believe that aspirin should be promoted instead of triflusal because of better evidence in the indications they share. Metamizole and triflusal are frequently used drugs in our country with limited long-term safety data and we believe that their use should be redirected to safer alternatives.

Our study excludes an overall risk of kidney function decline of NSAIDs in our population, possibly biased by caution in their prescription in this population. However, a possible detrimental effect of NSAIDs is not ruled out, in this sense, our results show a high risk with the high use of NSAIDs. It should also be noted that the use of paracetamol does not present a kidney function decline and therefore could be a safe drug in population with kidney function decline.

## Study limitations

One of the limitations of this study is that the data on drugs were obtained from billed prescriptions within the National Health Service, so adherence cannot be assured. Moreover, data on self-medication or drug prescription by private care providers are not available.

We cannot rule out a possible bias in population selection because case populations have a higher prevalence of cardiovascular pathology and could have a lower probability of receiving NSAIDs and in patients with initial reductions in eGFR, especially considering that decline in renal function is a continuous value (not a presence or absence value).

We have only considered eGFR to define decline in renal function, due to the absence of albuminuria data in nearly half of the patients. In addition, the date of analysis did not always coincide with the creatinine determination. We obtained and adjusted information for the main risk factors, except for proteinuria, as the value was not available in most patients. There may also be other confounding factors to consider.

The variable used (decline in renal function) from analytical tests available in the database could be less accurate in setting the index day compared with studies conducted directly on patients and there may be losses of information due to lack of recording of analytical results in setting the index day. There may also be a possible bias in the classification of both case and control due to the lack of available analytical values in the database. Another limitation of database studies could be the absence of register of non-pharmacological exposures that affect renal function.

## Conclusion

In our study, we observed a high risk of decline in renal function associated with metamizole and antiplatelet agents, especially triflusal and with high use of acetic acid derivatives NSAIDs, Coxibs, and major opioids. There was a low risk for acetaminophen and SYSADOA.

This high risk linked to metamizole is of particular concern, given its frequent use in our country as an alternative to NSAIDs. There is also concern about the high risk described by triflusal, as this drug is used for prevention or cardiovascular events. Further studies are necessary to confirm these results.

### Electronic supplementary material

Below is the link to the electronic supplementary material.

Supplementary Material 1

Supplementary Material 2

Supplementary Material 3

Supplementary Material 4

Supplementary Material 5

Supplementary Material 6

Supplementary Material 7

## Data Availability

The datasets generated and/or analysed during the present study are not publicly available because the Catalan Institute of Health is the data provider of SIDIAP, but are available from the corresponding author on reasonable request.

## Abbreviations

AAD: Acetic acid derivatives
ASA: Acetyl salicylic acid
ATC: Anatomical Therapeutic Chemical
CATSALUT: Catalan Health Service
CIs: Confidence Intervals
CKD: Chronic kidney disease
CKD-EPI: Chronic kidney disease epidemiology collaboration equation
Coxib: Selective cyclooxygenase-2 inhibitors
DDD: Defined daily doses
DM: Diabetes mellitus
ECAP: Electronic health records from primary care
eGFR: estimated Glomerular Filtration Rate
HT: Hypertension
ICD-10: International Statistical Classification of Diseases and Related Health Problems, 10th Revision
ICS: “Institut Català de la Salut”
NSAIDs: Non-steroidal anti-inflammatory drugs
OR: Odds ratio
Oxicam: Enolid acid derivatives NSAIDs
PAD: Propionic acid-derivatives
SIDIAP: Information System for the Development of Research in Primary Care
SYSADOA: Symptomatic Slow Action Drugs for Osteoarthritis

## References

[1] Charles C, Ferris AH. Chronic kidney disease. Prim Care. 2020;47(4):585–95. 10.1016/j.pop.2020.08.001. 33121630 10.1016/j.pop.2020.08.001

[2] Gorostidi M, Sánchez-Martínez M, Ruilope LM, Graciani A, de la Cruz JJ, Santamaría R, Del Pino MD, Guallar-Castillón P, de Álvaro F, Rodríguez-Artalejo F, Banegas JR. Chronic kidney disease in Spain: prevalence and impact of accumulation of cardiovascular risk factors. Nefrología. 2018;38(6):606–15. 10.1016/j.nefro.2018.04.004. 29914761 10.1016/j.nefro.2018.04.004

[3] Otero A, de Francisco A, Gayoso P, García F, EPIRCE Study Group. Prevalence of chronic renal disease in Spain: results of the EPIRCE study. Nefrologia. 2010;30(1):78–86. 10.3265/Nefrologia.pre2009.Dic.5732. 20038967 10.3265/Nefrologia.pre2009.Dic.5732

[4] Organització Catalana de Trasplantaments (OCATT). Registre de malalts renals de Catalunya, informe estadístic 2020. Departament de Salut, Generalitat de Catalunya 2022(35). https://trasplantaments.gencat.cat/web/.content/minisite/trasplantament/registres_activitat/registre_de_malalts_renals/arxius/Informe-RMRC-2020.pdf. Accessed 25 November 2022.

[5] De Broe ME, Elseviers MM. Over-the-counter analgesic use. J Am Soc Nephrol. 2009;20(10):2098–103. 10.1681/ASN.2008101097. 19423685 10.1681/ASN.2008101097

[6] Rexrode KM, Buring JE, Glynn RJ, Stampfer MJ, Youngman LD, Gaziano JM. Analgesic use and renal function in men. JAMA. 2001;286(3):315–21. 10.1001/jama.286.3.315. 11466097 10.1001/jama.286.3.315

[7] Perneger TV, Whelton PK, Klag MJ. Risk of kidney failure associated with the use of acetaminophen, aspirin, and nonsteroidal antiinflammatory drugs. N Engl J Med. 1994;331(25):1675–9. 10.1056/NEJM199412223312502. 7969358 10.1056/NEJM199412223312502

[8] Fored CM, Ejerblad E, Lindblad P, et al. Acetaminophen, aspirin, and chronic renal failure. N Engl J Med. 2001;345(25):1801–8. 10.1056/NEJMoa010323. 11752356 10.1056/NEJMoa010323

[9] Kurth T, Glynn RJ, Walker AM, et al. Analgesic use and change in kidney function in apparently healthy men. Am J Kidney Dis. 2003;42(2):234–44. 10.1016/s0272-6386(03)00647-4. 12900803 10.1016/s0272-6386(03)00647-4

[10] Ibáñez L, Morlans M, Vidal X, Martínez MJ, Laporte JR. Case-control study of regular analgesic and nonsteroidal anti-inflammatory use and end-stage renal disease. Kidney Int. 2005;67(6):2393–8. 10.1111/j.1523-1755.2005.00346.x. 15882284 10.1111/j.1523-1755.2005.00346.x

[11] Sciahbasi A, Arcieri R, Quarto M, et al. Impact of chronic aspirin and statin therapy on presentation of patients with acute myocardial infarction and impaired renal function. Prev Cardiol. 2010;13(1):18–22. 10.1111/j.1751-7141.2009.00050.x. 20021622 10.1111/j.1751-7141.2009.00050.x

[12] Curhan GC, Knight EL, Rosner B, Hankinson SE, Stampfer MJ. Lifetime nonnarcotic analgesic use and decline in renal function in women. Arch Intern Med. 2004;164(14):1519–24. 10.1001/archinte.164.14.1519. 15277282 10.1001/archinte.164.14.1519

[13] Gooch K, Culleton BF, Manns BJ, et al. NSAID use and progression of chronic kidney disease. Am J Med. 2007;120(3):280.e1-280.e2807. 10.1016/j.amjmed.2006.02.015. 10.1016/j.amjmed.2006.02.015 17349452

[14] Evans M, Fored CM, Bellocco R, et al. Acetaminophen, aspirin and progression of advanced chronic kidney disease. Nephrol Dial Transpl. 2009;24(6):1908–18. 10.1093/ndt/gfn745. 10.1093/ndt/gfn745 19155536

[15] Nderitu P, Doos L, Jones PW, Davies SJ, Kadam UT. Non-steroidal anti-inflammatory drugs and chronic kidney disease progression: a systematic review. Fam Pract. 2013;30(3):247–55. 10.1093/fampra/cms086. 23302818 10.1093/fampra/cms086

[16] Johnson AG, Nguyen TV, Day RO. Do nonsteroidal anti-inflammatory drugs affect blood pressure? A meta-analysis. Ann Intern Med. 1994;121(4):289–300. 10.7326/0003-4819-121-4-199408150-00011. 8037411 10.7326/0003-4819-121-4-199408150-00011

[17] Zhang J, Ding EL, Song Y. Adverse effects of cyclooxygenase 2 inhibitors on renal and arrhythmia events: meta-analysis of randomized trials. JAMA. 2006;296(13):1619–32. 10.1001/jama.296.13.jrv60015. 16968832 10.1001/jama.296.13.jrv60015

[18] Ficha técnica de etoricoxib. https://cima.aemps.es/cima/dochtml/ft/81465/FT_81465.html. Accessed 20 November 2022.

[19] Chan CC, Reid CM, Aw TJ, Liew D, Haas SJ, Krum H. Do COX-2 inhibitors raise blood pressure more than nonselective NSAIDs and placebo? An updated meta-analysis. J Hypertens. 2009;27(12):2332–41. 10.1097/HJH.0b013e3283310dc9. 19887957 10.1097/HJH.0b013e3283310dc9

[20] Dolati S, Tarighat F, Pashazadeh F, Shahsavarinia K, Gholipouri S, Soleimanpour H. The role of opioids in pain management in elderly patients with chronic kidney disease: a review article. Anesth Pain Med. 2020;10(5):e105754. 10.5812/aapm.105754. 34150565 10.5812/aapm.105754 PMC8207885

[21] Garg AX, Kurz A, Sessler DI, et al. Perioperative aspirin and clonidine and risk of acute kidney injury: a randomized clinical trial. JAMA. 2014;312(21):2254–64. 10.1001/jama.2014.15284. 25399007 10.1001/jama.2014.15284

[22] Palmer SC, Di Micco L, Razavian M, et al. Antiplatelet agents for chronic kidney disease. Cochrane Database Syst Rev. 2013;2CD008834. 10.1002/14651858.CD008834.pub2. 10.1002/14651858.CD008834.pub2 23450589

[23] Okada S, Morimoto T, Ogawa H, et al. Is long-term low-dose aspirin therapy associated with renal dysfunction in patients with type 2 diabetes? JPAD2 cohort study. PLoS ONE. 2016;11(1):e0147635. 10.1371/journal.pone.0147635. Published 2016 Jan 25. 26808136 10.1371/journal.pone.0147635 PMC4726501

[24] Nath KA. Platelets, antiplatelet therapy, and diabetic nephropathy. Mayo Clin Proc. 1988;63(1):80–85. 10.1016/s0025-6196(12)62670-6 10.1016/s0025-6196(12)62670-6 3275846

[25] Khajehdehi P, Roozbeh J, Mostafavi H. A comparative randomized and placebo-controlled short-term trial of aspirin and dipyridamole for overt type-2 diabetic nephropathy. Scand J Urol Nephrol. 2002;36(2):145–8. 10.1080/003655902753679454. 12028688 10.1080/003655902753679454

[26] Jansen MPB, Florquin S, Roelofs JJTH. The role of platelets in acute kidney injury. Nat Rev Nephrol. 2018;14(7):457–71. 10.1038/s41581-018-0015-5. 29760447 10.1038/s41581-018-0015-5

[27] Bolíbar B, Fina Avilés F, Morros R, et al. Base De Datos SIDIAP: la historia clínica informatizada de Atención primaria como fuente de información para la investigación epidemiológica [SIDIAP database: electronic clinical records in primary care as a source of information for epidemiologic research]. Med Clin (Barc). 2012;138(14):617–21. 10.1016/j.medcli.2012.01.020. 22444996 10.1016/j.medcli.2012.01.020

[28] García-Gil Mdel M, Hermosilla E, Prieto-Alhambra D, et al. Construction and validation of a scoring system for the selection of high-quality data in a Spanish population primary care database (SIDIAP). Inf Prim Care. 2011;19(3):135–45. 10.14236/jhi.v19i3.806. 10.14236/jhi.v19i3.806 22688222

[29] International Statistical Classification of Diseases and Related Health Problems 10th Revision. World Health Organitzation. https://icd.who.int/browse10/2019/en#/. Accessed 25 November 2022.

[30] Earley A, Miskulin D, Lamb EJ, Levey AS, Uhlig K. Estimating equations for glomerular filtration rate in the era of creatinine standardization: a systematic review. Ann Intern Med. 2012;156(11):785–95. 10.7326/0003-4819-156-6-201203200-00391. 22312131 10.7326/0003-4819-156-6-201203200-00391

[31] World Health Organization Collaborating Center for Drug Statistics Methodology. Anatomical Therapeutic Chemical (ATC) Classifica-tion System. http://www.whocc.no/atc_ddd_index/. Accessed 12 August 2021.

[32] Castellsague J, Riera-Guardia N, Calingaert B, et al. Individual NSAIDs and upper gastrointestinal complications: a systematic review and meta-analysis of observational studies (the SOS project). Drug Saf. 2012;35(12):1127–46. 10.2165/11633470-000000000-00000. 23137151 10.2165/11633470-000000000-00000 PMC3714137

[33] Oscanoa-Espinoza T, Lizaraso-Soto F. Antiinflamatorios no esteroides: seguridad gastrointestinal, cardiovascular y renal [Nonsteroidal antiinflammatory drugs: gastrointestinal and cardiovascular and renal safety]. Rev Gastroenterol Peru. 2015;35(1):63–71. PMID: 25875519. 25875519

[34] McGettigan P, Henry D. Cardiovascular risk with non-steroidal anti-inflammatory drugs: systematic review of population-based controlled observational studies. PLoS Med. 2011;8(9):e1001098. 10.1371/journal.pmed.1001098. 21980265 10.1371/journal.pmed.1001098 PMC3181230

[35] McGettigan P, Henry D. Use of non-steroidal anti-inflammatory drugs that elevate cardiovascular risk: an examination of sales and essential medicines lists in low-. middle-. And high-income countries. PLoS Med. 2013;10(2):e1001388. 10.1371/journal.pmed.1001388. 23424288 10.1371/journal.pmed.1001388 PMC3570554

[36] Hinz B, et al. Dipyrone elicits substantial inhibition of peripheral cyclooxygenases in humans: new insights into the pharmacology of an old analgesic. FASEB J 2007;21:2343–51. 10.1096/fj.06-8061com. 10.1096/fj.06-8061com 17435173

[37] Blaser LS et al. Sep. Comparative Effects of Metamizole (Dipyrone) and Naproxen on Renal Function and Prostacyclin Synthesis in Salt-Depleted Healthy Subjects - A Randomized Controlled Parallel Group Study. Front Pharmacol 2021;12:620635. 10.3389/fphar.2021.620635 10.3389/fphar.2021.620635 PMC8453264 34557087

[38] Stueber T, Buessecker L, Leffler A, Gillmann Stueber HJ. The use of dipyrone in the ICU is associated with acute kidney injury: a retrospective cohort analysis. Eur J Anaesthesiol vol. 2017;34(10):673–80. 10.1097/EJA.0000000000000627. 10.1097/EJA.0000000000000627 28306590

[39] Pontes C, Marsal JR, Elorza JM, Aragón M, Prieto-Alhambra D, Morros R. Analgesic use and risk for Acute coronary events in patients with osteoarthritis: a population-based, nested case-control study. Clin Ther. 2018;40(2):270–83. 10.1016/j.clinthera.2017.12.011. 29398161 10.1016/j.clinthera.2017.12.011

[40] Palmer SC, Di Micco L, Razavian M, Antiplatelet agents for chronic kidney disease. Cochrane Database Syst Rev. 2013;(2):CD008834. 10.1002/14651858.CD008834.pub2 10.1002/14651858.CD008834.pub2 23450589

[41] Su X, Yan B, Wang L, Lv J, Cheng H, Chen Y. Effect of antiplatelet therapy on cardiovascular and kidney outcomes in patients with chronic kidney disease: a systematic review and meta-analysis. BMC Nephrol. 2019;20(1):309. 10.1186/s12882-019-1499-3 10.1186/s12882-019-1499-3 PMC6686545 31390997

[42] Poley A, Ortega JA, Medregal M, Martín M, Hermosilla C, Mora F. Prevalencia de enfermedades osteoarticulares y consumo de recursos. Calidad de vida y dependencia en pacientes con artrosis. Semergen. 2011;37(9):462–467. https://www.elsevier.es/es-revista-medicina-familia-semergen-40-articulo-prevalencia-enfermedades-osteoarticulares-consumo-recursos--S113835931100147X. Accessed 25 November 2022.

[43] Utilización de antiinflamatorios no esteroides (AINE) en España. 1992–2006. Agencia española de medicamentos y productos sanitarios 2014. https://www.aemps.gob.es/medicamentosUsoHumano/observatorio/docs/AINE.pdf. Accessed 25 November 2022.

[44] Utilización de medicamentos antiinflamatorios no esteroideos en España durante el periodo 2013–2016. Agencia española de medicamentos y productos sanitarios 2017. https://www.aemps.gob.es/medicamentosUsoHumano/observatorio/docs/antiinflamatorios-AINEs-periodo-2013-2016.pdf. Accessed 25 November 2022.

