# Supplementary figures and images for: NSAIDs, analgesics, antiplatelet drugs, and decline in renal function: a retrospective case-control study with SIDIAP database
Source: BMC Pharmacol Toxicol. 2024 Aug 28;25:58. doi: 10.1186/s40360-024-00771-5 (PMC11351315; doi:10.1186/s40360-024-00771-5)

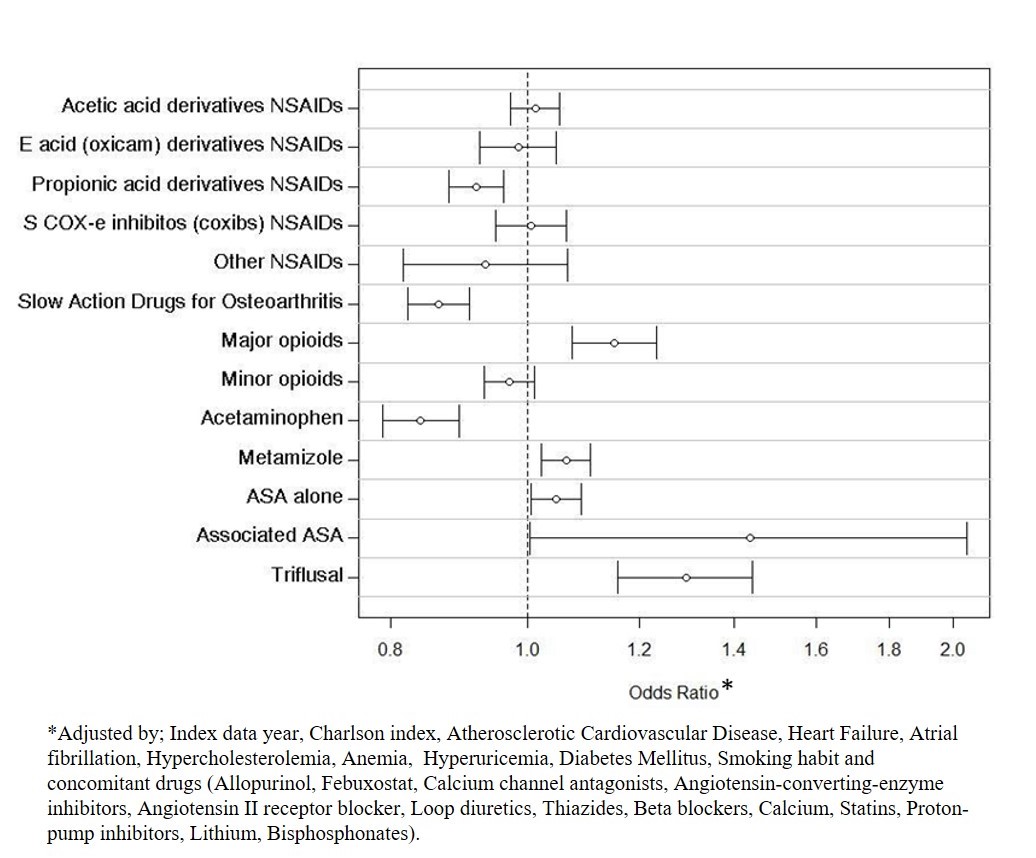

Supplement: Supplementary file 1 — Supplementary Material 1 [file 40360_2024_771_MOESM1_ESM.jpg]

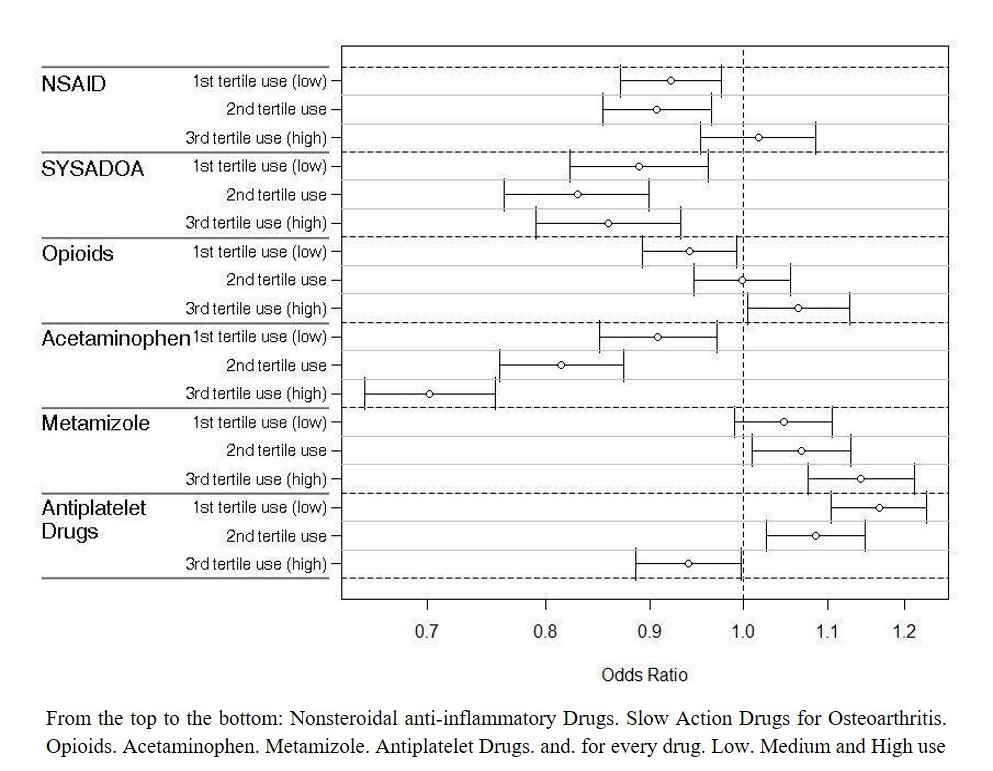

Supplement: Supplementary file 2 — Supplementary Material 2 [file 40360_2024_771_MOESM2_ESM.jpg]
