# Supplementary material for: NSAIDs, analgesics, antiplatelet drugs, and decline in renal function: a retrospective case-control study with SIDIAP database
Source: BMC Pharmacol Toxicol. 2024 Aug 28;25:58. doi: 10.1186/s40360-024-00771-5 (PMC11351315; doi:10.1186/s40360-024-00771-5)
Supplement: Supplementary file 3 — Supplementary Material 3 [file 40360_2024_771_MOESM3_ESM.docx]

**Supplementary Table 1.** Study baseline covariates and excluded diseases closely related to the possibility of presenting CKD.

| **Study baseline covariates** | |
| --- | --- |
| Charlson | No or low comorbidity (0 to 2 points)  High comorbidity (3 or more points) |
| Atherosclerotic Cardiovascular Disease (ICD-10) | I20, I21, I21.0, I21.1, I21.2, I21.3, I21.4, I21.9, I22, I22.0, I22.1, I22.8, I22.9, I23, I23.0, I23.1, I23.2, I23.3, I23.4, I23.5, I23.6, I23.8 I24, I25 I63, I64, I67.8, I67.9, I69, G45, G46, 170, I73 |
| Heart Failure (ICD-10) | I11.0, I13.0, I13.2, I50, I50.0, I50.1, I50.9, I50.90, I50.91 |
| Atrial fibrillation (ICD-10) | I48, I49.0 |
| Hypercholesterolemia (ICD-10) | E78.0,E78.1,E78.2, E78.4, E78.5, E78.8, E78.9 |
| Anemia (ICD-10) | D50, D51, D52, D53, D55, D56, D57, D58, D59, D60, D61, D62, D63, D64 |
| Hyperuricemia (ICD-10) | E79, M10, M10.0, M10.1, M10.2, M10.3, M10.4, M10.90 |
| Type 2 Diabetes Mellitus (ICD-10) | E10,E11, E12, E13, E14, N08.3 |
| Hypertension (ICD-10) | I10, I11, I11.0, I11.9, I12, I12.0, I12.9, I13, I13.0, I13.1, I13.2, I13.9, I15, I15.0, I15.1, I15.2, I15.8, I15.9 |
| **Diseases closely related to the possibility of presenting CKD** (ICD-10) | |
| **Renal conditions** | |
| Acute nephritic Syndrome | N00 |
| Rapidly progressive nephritic Syndrome | N01 |
| Recurring and persistent hematury | N02 |
| Chronic nephritic Syndrome | N03 |
| Nephrotic Syndrome | N04 |
| Non-specific nephritic Syndrome | N05 |
| Terminal kidney failure | N18.0 |
| Cystic disease of the Kidney | Q61 |
| Failure and rejection of kidney transplantation | T86.1 |
| Extracorporeal dialysis | Z49.1 |
| **Rheumatological diseases** | |
| Rheumatoid arthritis | M05 |
| Systemic lupus erythematosus, | M32 |
| Amyloidosis, | E85 |
| Ankylosing spondylitis, | M45 |
| Systemic sclerosis, | M34 |
| Polyarteritis nodosa | M30 |
| Other necrotizing vasculopathies | M31 |
| Other systemic disorders of the connective tissue | M35, M36 |
| Dermatopolymyositis, | M33 |
| Polyarteritis nodosa | M30 |
| Sarcoidosis | D86 |
| **Oncological pathology** | C00, C01, C02, C03, C04, C05, C06, C07, C08, C09, C10, C11, C12, C13, C14, C15, C16, C17, C18, C19, C20, C21, C22 ,C23, C24, C25, C26, C30, C31, C32, C33, C34, C37, C38, C39, C40, C41, C43, C44, C45, C46, C47, C48, C49, C50, C51, C52, C53, C54, C55, C56, C57, C58, C60, C61, C62, C63, C64, C65, C66, C67, C68, C69, C70, C71, C72, C73, C74, C75, C76, C77, C78, C79, C80, C81, C82, C83, C84, C85, C88, C90, C91, C92, C93, C94, C95, C96, C97, |
| **Patients with transplantation** | |
| Renal transplantation | Z94.0 |
| Hepatic transplantation | Z94.4 |
| Cardiac transplantation | Z94.1 |
| Pulmonary transplantation | Z94.2 |
| Pancreatic transplantation | Z94.8 |
| Cardiac and pulmonary transplantation | Z94.3 |
