# Supplementary material for: NSAIDs, analgesics, antiplatelet drugs, and decline in renal function: a retrospective case-control study with SIDIAP database
Source: BMC Pharmacol Toxicol. 2024 Aug 28;25:58. doi: 10.1186/s40360-024-00771-5 (PMC11351315; doi:10.1186/s40360-024-00771-5)
Supplement: Supplementary file 4 — Supplementary Material 4 [file 40360_2024_771_MOESM4_ESM.docx]

**Supplementary Table 2**. Related drugs to renal involvement and Study Drugs

| **Drug related to renal involvement** | |
| --- | --- |
| - Angiotensin-converting-enzyme inhibitors and/or angiotensin II receptor blocker (C09) - Calcium channel antagonists (C08), beta-blockers (C07) - Loop diuretics (C03) - Thiazides (C03A, C03B, C03E) - Statins (C10AA) - Calcium (A12A) - Proton-pump inhibitors (A02BC) - Lithium (N05AN) - Bisphosphonates (M05BA) - Allopurinol (M04AA01) - Febuxostat (M04AA03)) | |
| **Study Drugs** | |
| Drug groups | ATC codes Study Drugs: |
| NSAIDs | M01AA: [Butylpyrazolidines](https://prvademecum.es/app/atcs/view/M01AA/butilpirazolidinas#M01AA)  M01AB:[Acetic acid derivatives](https://prvademecum.es/app/atcs/view/M01AA/butilpirazolidinas#M01AB)  M01AC: [Oxicams](https://prvademecum.es/app/atcs/view/M01AA/butilpirazolidinas#M01AC)  M01AE:[Propionic acid derivatives](https://prvademecum.es/app/atcs/view/M01AA/butilpirazolidinas#M01AE)  M01AX0:[Other anti-inflammatory and anti-rheumatic agents, non-steroids](https://prvademecum.es/app/atcs/view/M01AA/butilpirazolidinas#M01AX)  M01AG: Fenamates  M01AH Coxibs |
| Symptomatic Slow Action Drugs for Osteoarthritis –SYSADOA | M01AX05 Glucosamine  M01AX25 Chondroitin,  M01AX21 Diacerein |
| Opioid analgesics | N02A |
| Non-opioid analgesics (Acetaminophen and metamizole) | N02B |
| Antiplatelet agents | B01AC |
