# Supplementary material for: NSAIDs, analgesics, antiplatelet drugs, and decline in renal function: a retrospective case-control study with SIDIAP database
Source: BMC Pharmacol Toxicol. 2024 Aug 28;25:58. doi: 10.1186/s40360-024-00771-5 (PMC11351315; doi:10.1186/s40360-024-00771-5)
Supplement: Supplementary file 5 — Supplementary Material 5 [file 40360_2024_771_MOESM5_ESM.docx]

**Supplementary Table 3. Multivariate regression model on adjusted decline in renal function by cumulative doses of drug subgroups (categorized in DDD tertiles)**

|  |  | **Adjusted**  **OR (95%CI)** | **p-value** |
| --- | --- | --- | --- |
| Acetic acid derivatives NSAIDs | *No use* | (ref.) |  |
|  | *Low use* | 0.97 (0.92, 1.02) | 0.200 |
|  | *Medium use* | 1.01 (0.96, 1.07) | 0.683 |
|  | *High use* | 1.09 (1.03, 1.15) | 0.003 |
| Enolic acid (oxicam) derivatives NSAIDs | *No use* | (ref.) |  |
|  | *Low use* | 0.95 (0.87, 1.03) | 0.234 |
|  | *Medium use* | 0.98 (0.87, 1.11) | 0.783 |
|  | *High use* | 1.01 (0.91, 1.13) | 0.863 |
| Propionic acid derivatives NSAIDs | *No use* | (ref.) |  |
|  | *Low use* | 0.94 (0.90, 0.99) | 0.023 |
|  | *Medium use* | 0.91 (0.86, 0.96) | 0.001 |
|  | *High use* | 0.94 (0.89, 1.00) | 0.043 |
| Coxibs NSAIDs | *No use* | (ref.) |  |
|  | *Low use* | 0.87 (0.80, 0.95) | 0.001 |
|  | *Medium use* | 1.00 (0.91, 1.11) | 0.946 |
|  | *High use* | 1.19 (1.08, 1.30) | <0.001 |
| Other NSAIDs | *No use* | (ref.) |  |
|  | *Low use* | 0.85 (0.71, 1.01) | 0.072 |
|  | *Medium use* | 1.00 (0.71, 1.38) | 0.977 |
|  | *High use* | 1.08 (0.84, 1.37) | 0.548 |
| Major opioids | *No use* | (ref.) |  |
|  | *Low use* | 1.09 (0.98, 1.23) | 0.124 |
|  | *Medium use* | 1.15 (1.03, 1.29) | 0.014 |
|  | *High use* | 1.15 (1.03, 1.29) | 0.013 |
| Minor opioids | *No use* | (ref.) |  |
|  | *Low use* | 0.95 (0.90, 1.00) | 0.047 |
|  | *Medium use* | 1.00 (0.95, 1.05) | 0.938 |
|  | *High use* | 1.03 (0.97, 1.10) | 0.272 |
| ASA alone | *No use* | (ref.) |  |
|  | *Low use* | 1.16 (1.10, 1.22) | <0.001 |
|  | *Medium use* | 1.06 (1.00, 1.12) | 0.046 |
|  | *High use* | 0.92 (0.87, 0.98) | 0.008 |
| Associated ASA | *No use* | (ref.) |  |
|  | *Low use* | 1.28 (0.71, 2.26) | 0.397 |
|  | *Medium use* | 1.09 (0.57, 2.06) | 0.785 |
|  | *High use* | 1.87 (0.99, 3.53) | 0.054 |
| Triflusal | *No use* | (ref.) |  |
|  | *Low use* | 0.98 (0.80, 1.20) | 0.865 |
|  | *Medium use* | 1.23 (1.02, 1.48) | 0.026 |
|  | *High use* | 1.06 (0.92, 1.21) | 0,445 |

*Adjusted by; Index data year, Charlson index, Atherosclerotic Cardiovascular Disease, Heart Failure, Atrial fibrillation, Hypercholesterolemia, Anemia, Hyperuricemia, Diabetes Mellitus, Smoking habit and concomitant drugs (Allopurinol, Febuxostat, Calcium channel antagonists, Angiotensin-converting-enzyme inhibitors, Angiotensin II receptor blocker, Loop diuretics, Thiazides, Beta blockers, Calcium, Statins, Proton-pump inhibitors, Lithium, Bisphosphonates)
