# Supplementary material for: NSAIDs, analgesics, antiplatelet drugs, and decline in renal function: a retrospective case-control study with SIDIAP database
Source: BMC Pharmacol Toxicol. 2024 Aug 28;25:58. doi: 10.1186/s40360-024-00771-5 (PMC11351315; doi:10.1186/s40360-024-00771-5)
Supplement: Supplementary file 6 — Supplementary Material 6 [file 40360_2024_771_MOESM6_ESM.docx]

**Supplementary Table 4. Multivariate regression model on adjusted decline in renal function by pattern of drug groups use**

|  |  | **Adjusted**  **OR (95%CI)** | **p-value** |
| --- | --- | --- | --- |
| Nonsteroidal anti-inflammatory Drugs | *No use* | (ref.) |  |
|  | *Recent use* | 0.97 (0.92, 1.03) | 0.343 |
|  | *Remote use* | 0.91 (0.86, 0.96) | <0.001 |
| Slow Action Drugs for Osteoarthritis | *No use* | (ref.) |  |
|  | *Recent use* | 0.82 (0.73, 0.91) | <0.001 |
|  | *Remote use* | 0.88 (0.83, 0.93) | <0.001 |
| Opioids | *No use* | (ref.) |  |
|  | *Recent use* | 1.04 (0.98, 1.10) | 0.163 |
|  | *Remote use* | 0.96 (0.92, 1.01) | 0.080 |
| Acetaminophen | *No use* | (ref.) |  |
|  | *Recent use* | 0.80 (0.74, 0.85) | <0.001 |
|  | *Remote use* | 0.89 (0.83, 0.95) | <0.001 |
| Metamizole | *No use* | (ref.) |  |
|  | *Recent use* | 1.22 (1.15, 1.29) | <0.001 |
|  | *Remote use* | 1.01 (0.97, 1.06) | 0.653 |
| Antiplatelet Drugs | *No use* | (ref.) |  |
|  | *Recent use* | 1.08 (1.03, 1.13) | 0.001 |
|  | *Remote use* | 1.06 (1.00, 1.13) | 0.035 |

*Adjusted by; Index data year, Charlson index, Atherosclerotic Cardiovascular Disease, Heart Failure, Atrial fibrillation, Hypercholesterolemia, Anemia, Hyperuricemia, Diabetes Mellitus, Smoking habit and concomitant drugs (Allopurinol, Febuxostat, Calcium channel antagonists, Angiotensin-converting-enzyme inhibitors, Angiotensin II receptor blocker, Loop diuretics, Thiazides, Beta blockers, Calcium, Statins, Proton-pump inhibitors, Lithium, Bisphosphonates)
