# Supplementary material for: NSAIDs, analgesics, antiplatelet drugs, and decline in renal function: a retrospective case-control study with SIDIAP database
Source: BMC Pharmacol Toxicol. 2024 Aug 28;25:58. doi: 10.1186/s40360-024-00771-5 (PMC11351315; doi:10.1186/s40360-024-00771-5)
Supplement: Supplementary file 7 — Supplementary Material 7 [file 40360_2024_771_MOESM7_ESM.docx]

**Supplementary Table 5. Multivariate regression model on adjusted decline in renal function by pattern of drug subgroups use**

|  |  | **Adjusted**  **OR (95%CI)** | **p-value** |
| --- | --- | --- | --- |
| Acetic acid derivatives NSAIDs | *No use* | (ref.) |  |
|  | *Recent use* | 1.18 (1.11, 1.26) | <0.001 |
|  | *Remote use* | 0.97 (0.93, 1.01) | 0.191 |
| Enolic acid (oxicam) derivatives NSAIDs | *No use* | (ref.) |  |
|  | *Recent use* | 0.97 (0.78, 1.20) | 0.802 |
|  | *Remote use* | 0.99 (0.93, 1.05) | 0.709 |
| Propionic acid derivatives NSAIDs | *No use* | (ref.) |  |
|  | *Recent use* | 0.94 (0.89, 0.99) | 0.017 |
|  | *Remote use* | 0.92 (0.88, 0.97) | <0.001 |
| Coxibs NSAIDs | *No use* | (ref.) |  |
|  | *Recent use* | 1.14 (1.03, 1.27) | 0.013 |
|  | *Remote use* | 0.96 (0.90, 1.03) | 0.250 |
| Other NSAIDs | *No use* | (ref.) |  |
|  | *Recent use* | 0.69 (0.47, 1.00) | 0.055 |
|  | *Remote use* | 0.98 (0.85, 1.13) | 0.771 |
| Slow Action Drugs for Osteoarthritis | *No use* | (ref.) |  |
|  | *Recent use* | 0.81 (0.73, 0.90) | <0.001 |
|  | *Remote use* | 0.88 (0.83, 0.93) | <0.001 |
| Major opioids | *No use* | (ref.) |  |
|  | *Recent use* | 1.16 (1.06, 1.27) | 0.001 |
|  | *Remote use* | 1.11 (1.01, 1.22) | 0.031 |
| Minor opioids | *No use* | (ref.) |  |
|  | *Recent use* | 1.01 (0.95, 1.07) | 0.845 |
|  | *Remote use* | 0.97 (0.92, 1.01) | 0.111 |
| Acetaminophen | *No use* | (ref.) |  |
|  | *Recent use* | 0.80 (0.75, 0.85) | <0.001 |
|  | *Remote use* | 0.89 (0.83, 0.95) | <0.001 |
| Metamizole | *No use* | (ref.) |  |
|  | *Recent use* | 1.20 (1.14, 1.28) | <0.001 |
|  | *Remote use* | 1.01 (0.97, 1.06) | 0.643 |
| ASA alone | *No use* | (ref.) |  |
|  | *Recent use* | 1.06 (1.01, 1.11) | 0.013 |
|  | *Remote use* | 1.06 (1.00, 1.13) | 0.040 |
| Associated ASA | *No use* | (ref.) |  |
|  | *Recent use* | 1.64 (1.07, 2.50) | 0.023 |
|  | *Remote use* | 1.05 (0.54, 1.97) | 0.887 |
| Triflusal | *No use* | (ref.) |  |
|  | *Recent use* | 1.89 (1.58, 2.25) | <0.001 |
|  | *Remote use* | 1.06 (0.92, 1.21) | 0.445 |

*Adjusted by; Index data year, Charlson index, Atherosclerotic Cardiovascular Disease, Heart Failure, Atrial fibrillation, Hypercholesterolemia, Anemia, Hyperuricemia, Diabetes Mellitus, Smoking habit and concomitant drugs (Allopurinol, Febuxostat, Calcium channel antagonists, Angiotensin-converting-enzyme inhibitors, Angiotensin II receptor blocker, Loop diuretics, Thiazides, Beta blockers, Calcium, Statins, Proton-pump inhibitors, Lithium, Bisphosphonates)
